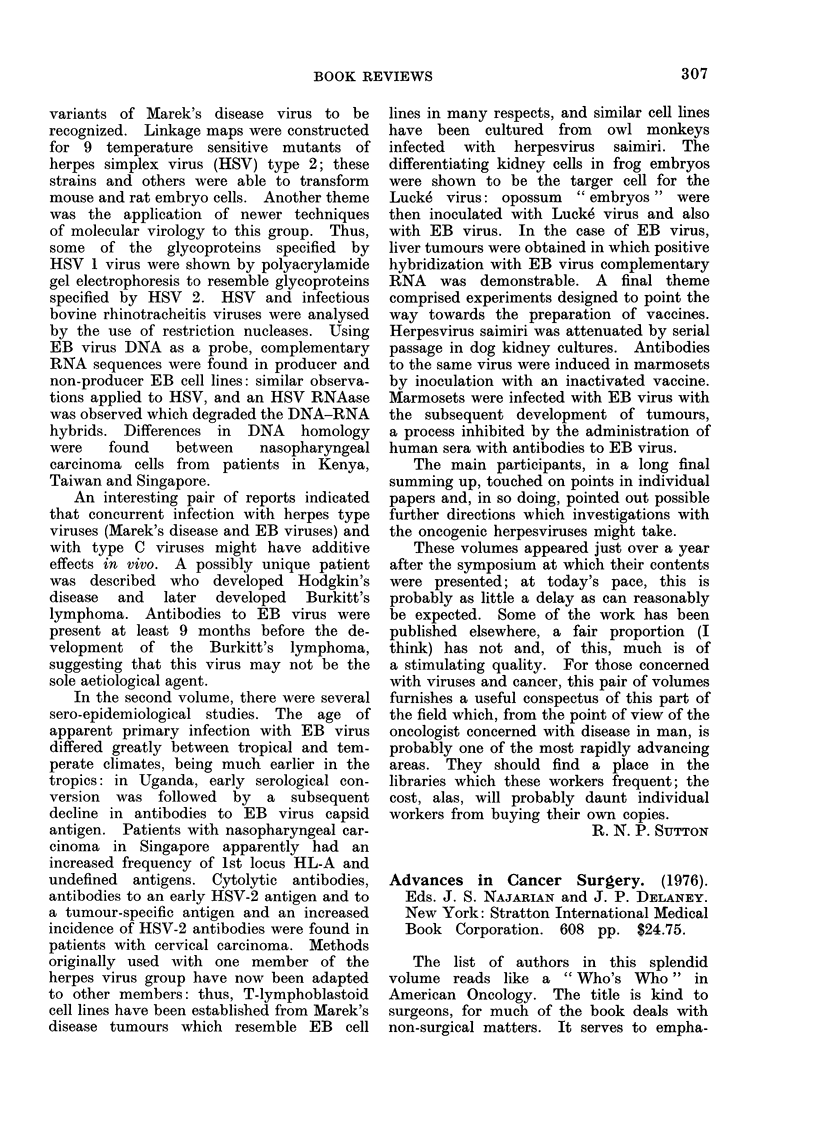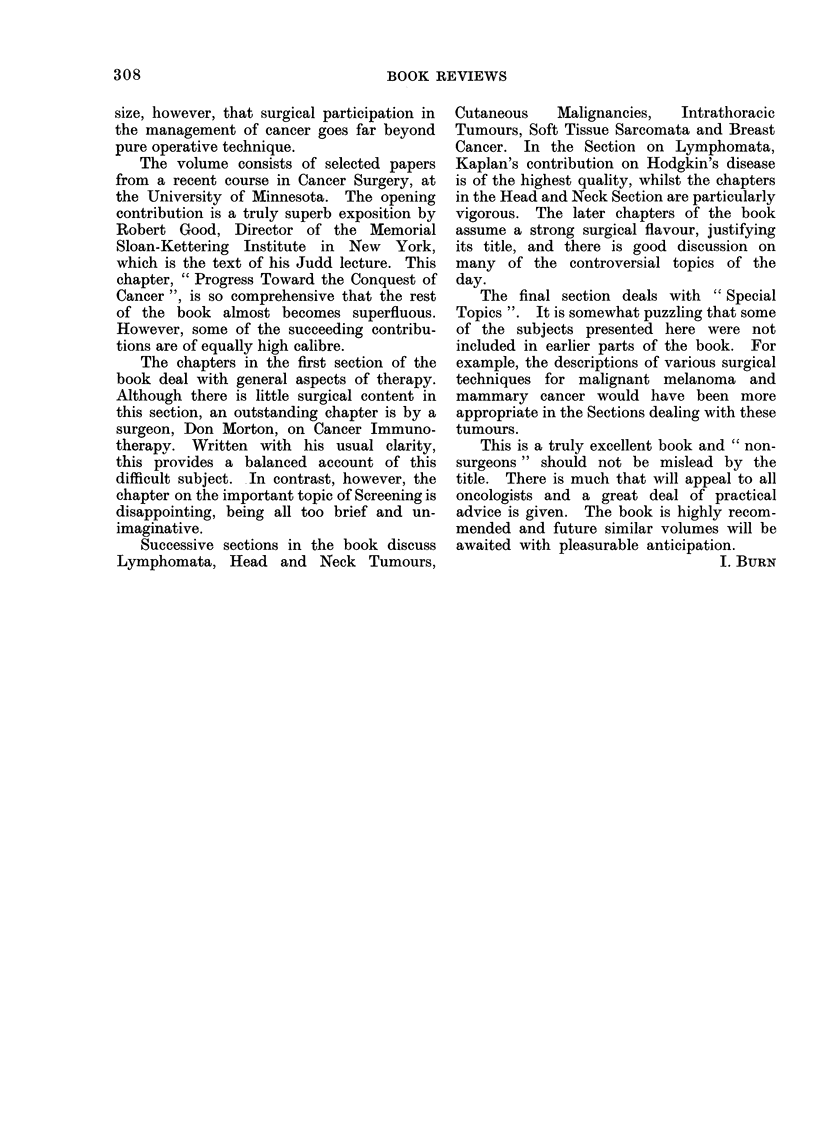# Advances in Cancer Surgery

**Published:** 1976-09

**Authors:** I. Burn


**Advances in Cancer Surgery.** (1976). Eds. J. S. Najarian and J. P. Delaney. New York: Stratton International Medical Book Corporation. 608 pp. $24.75.

The list of authors in this splendid volume reads like a " Who's Who " in American Oncology. The title is kind to surgeons, for much of the book deals with non-surgical matters. It serves to emphasize, however, that surgical participation in the management of cancer goes far beyond pure operative technique.

The volume consists of selected papers from a recent course in Cancer Surgery, at the University of Minnesota. The opening contribution is a truly superb exposition by Robert Good, Director of the Memorial Sloan-Kettering Institute in New York, which is the text of his Judd lecture. This chapter, " Progress Toward the Conquest of Cancer ", is so comprehensive that the rest of the book almost becomes superfluous. However, some of the succeeding contributions are of equally high calibre.

The chapters in the first section of the book deal with general aspects of therapy. Although there is little surgical content in this section, an outstanding chapter is by a surgeon, Don Morton, on Cancer Immunotherapy. Written with his usual clarity, this provides a balanced account of this difficult subject. In contrast, however, the chapter on the important topic of Screening is disappointing, being all too brief and unimaginative.

Successive sections in the book discuss Lymphomata, Head and Neck Tumours, Cutaneous Malignancies, Intrathoracic Tumours, Soft Tissue Sarcomata and Breast Cancer. In the Section on Lymphomata, Kaplan's contribution on Hodgkin's disease is of the highest quality, whilst the chapters in the Head and Neck Section are particularly vigorous. The later chapters of the book assume a strong surgical flavour, justifying its title, and there is good discussion on many of the controversial topics of the day.

The final section deals with " Special Topics ". It is somewhat puzzling that some of the subjects presented here were not included in earlier parts of the book. For example, the descriptions of various surgical techniques for malignant melanoma and mammary cancer would have been more appropriate in the Sections dealing with these tumours.

This is a truly excellent book and " non-surgeons " should not be mislead by the title. There is much that will appeal to all oncologists and a great deal of practical advice is given. The book is highly recommended and future similar volumes will be awaited with pleasurable anticipation.

I. Burn